# Priority setting in research: user led mental health research

**DOI:** 10.1186/s40900-016-0054-7

**Published:** 2017-02-01

**Authors:** Marjorie Ghisoni, Christine Ann Wilson, Karen Morgan, Bethan Edwards, Natalie Simon, Emma Langley, Helen Rees, Amanda Wells, Philip John Tyson, Phil Thomas, Allen Meudell, Frank Kitt, Brian Mitchell, Alan Bowen, Jason Celia

**Affiliations:** 1 School of Health Care Sciences, Bangor University, Fron Heulog, Ffriddoedd Road, Bangor, Gwynedd, North Wales LL57 2EF UK; 2 Hafal, Unit B3, Lakeside technology Park, Phoenix Way, Llansamlet, Swansea, Wales SA79FE UK; 3 Mental Health Service User Involvement Officer, Gwent Association of Voluntary Organisations, Ty Derwen, Church Road, Newport, South Wales NP19 7EJ UK; 4 80,Penallta Road, Ystrad Mynach, Hengoed, Wales CF82 7BG UK; 5 Health and Care Research Wales Support Centre, Castlebridge 4, 15-19 Cowbridge Road East, Cardiff, CF11 9AB Wales, UK; 6 Cardiff, South Wales UK; 7 6 Hafan Deg, Llanfair Caereinion, Powys Wales SY21 0RU UK; 8 University of South Wales, Treforest Campus, Pontypridd, Wales CF37 1DL UK; 9 National Centre for Mental Health, Hadyn Ellis Building, Maindy Road, Cardiff, CF24 4HQ UK

**Keywords:** Delivery of health services, Public health, Patient and public involvement, Mental health, Research, User involvement, User priorities, Priority setting

## Abstract

**Plain English summary:**

Involving people in health research is increasingly recognised as being important to make sure that research is focused more on the needs of people who use health services. At present, ideas about what should be researched most often comes from researchers and/or health professionals like doctors and nurses rather than people with a lived experience of mental illness. In this study, we will talk with this group of people from across Wales to explore what they think research into their health services should focus on. The findings from this work will help to influence the work of the National Centre for Mental Health Research Partnership Group; as well as` researchers and health professionals and others who concentrate on mental health research. The Research group is a partnership between people with a lived experience of mental ill health and professionals with an interest in mental ill health. The group plan to take forward the ideas that came from this research and some of the ideas have already been used to increase funding in the area of mental health research.

**Abstract:**

**Background** This paper is the result of continued collaboration between members of the Service User and Carer Research Partnership, based in Wales and supported by the National Centre for Mental Health, Health and Care Research Wales, and Hafal. The aim of this study was to explore the research priorities of people with experience of mental health services which include people with a lived experience of mental ill health, their carers, and professionals.

**Method** A nominal group technique was used to gather data. A one-day workshop ‘Getting Involved in Research: Priority Setting’ was held to gather the ideas and suggestions for research priorities from people who have experience of mental health services.

**Results** Twenty-five participants attended the workshop. 5 were mental health professionals, 20 had a lived experience of mental ill health, (of which 3 were also carers). 11 were male and 14 were female. 120 research ideas were generated across 6 ‘Ideas Generating Workstations’. Participants took part in a 3 stage vote to narrow down the ideas to 2 main research priorities.

**Conclusion** The two main research priority areas that were identified:

1. ‘Developing the knowledge of mental health issues amongst school-aged children’ as a vehicle to overcome stigma and discrimination, and to support young people to manage their own mental health.
2. ‘Developing education as a tool for recovery’, for example by peer support. In addition, participants engaged in a notable discussion over the research priority: ‘How are carers supported during the recovery of the person for whom they care?’

## Background

This paper is the result of continued collaboration between members of the Service User and Carer Partnership Research Development Group, which, following the reconfiguration of the Research Development Groups (RDG’s) in Wales, became the Service User and Carer Research Partnership; supported, by the National Centre for Mental Health (NCMH), Health Care Research Wales and Hafal. The group is comprised of people with lived experience of mental health issues, mental health professionals, and academics [1]. One of the key aims of this group is to conduct genuine, user-led mental health research that is relevant and meaningful to all the members, (which is not academically-led) [2, 3]. The future of user-led research in mental health will therefore be discussed further on in this paper.

The Mental Health Research Network Cymru (MHRN-C) was formed in 2006 and included the Service User and Carer Partnership Research Development Group (SUCPRDG) which was set up by people with experience of mental health issues and their supporters, to encourage and enable input of members into the MHRN-C and wider research [1]. MHRN-C was a Registered Research Group (RRG) within the National Institute of Social Care and Health Research (NISCHR) funded infrastructure up until April 2015, when it went through reorganisation. As part of the new Health and Care Research Wales infrastructure the SUCPRDG was adopted by the National Centre for Mental Health (NCMH) and continues to support public engagement, involvement and participation within this group. During this period of change, members of the group have explored their own roles and terms used to describe what they do. The following discussion about the terminology used was carried out to ensure that all members were working in collaboration towards shared outcomes.

### Terms for ‘service user’ (people with a lived experience of mental ill health)

Before the research took place a collaborative discussion was held around the use of the term ‘Service User’, which was disliked by many members of the group. Members of the group both with lived experience and academics offered alternative suggestions for the term. We found that there was no consensus on terms used when describing public involvement in research. Barnes and Cotterell [3] would suggest that language is not neutral and descriptive nouns such as Service User, Lay Person, Consumer, and Patient may, for some, have negative connotations and imply misleading roles [4–7], while others may perceive a more positive image. Members acknowledged this problem, but also expressed their dislike of the term ‘service user’ to describe people who are experiencing, or have experienced, mental ill health. We felt this was outdated and denigrating and should be replaced. After some face to face discussions, a call went out by email to all members to send in their ideas for a more acceptable term. ‘Service expert’ had previously been considered and ‘mental health survivor’ and ‘person with lived experience of mental health services’ were added to the list of possibilities. Contributors to this paper decided on the term ‘people with experience of mental health issues’ as this was suggested by the highest number of members. Participants in the workshop will thus be referred to in this way in the rest of this paper.

### Reviewing the literature on the need for people with experience of mental health services engagement in research

Effective research and development (R&D) strategies require well-informed research priorities based on evidence of need [8, 9]. Public involvement in research prioritisation provides a route to identifying relevant and quality research topics that complement other methods of focusing research and development, (R&D) activity [10]. Patients may have different research priorities from clinicians or academics [11–13]. For example, in a study by [14] people with experience of mental health issues were more interested in research that was social and psychological rather than biomedical Another such factor is linked to the status of the participants: with those of a higher status or a status in which their opinions may be perceived to carry more weight. An example of this could be where a professional has a completely different set of priorities from people with a perceived lower status (such as those with a lived experience); and use that status to claim legitimacy, whilst at the same time calling into question the legitimacy of the lower status participants [15].

However, many people have participated in consultations to identify research themes incorporating their views [16].

As suggested in public involvement literature [17], research should be ‘with’ or ‘by’ members of the public, rather than ‘to’, ‘about’ and ‘for’ them. In addition, early involvement in the research process encourages people with experience of mental health issues to feel a sense of ownership [17], refocuses research in line with patient and carer concerns, initiates and accelerates research ideas [18], and can make research applications more fundable [19]. Responding to public and patient priorities for research can also make the research process more relevant and efficient, helping to reduce waste in the production and reporting of research evidence [20]. Systematic reviews suggest the quality and appropriateness of research can be improve when people with experience of mental ill health are involved. For example, their inclusion helps to embed research findings into real-world settings [18, 21]. United Kingdom policy recommends that people who use services should be involved in all stages of research, from identifying research topics to disseminating findings [22–26]. Most national and international research funding programmes expect patients and carers to be involved in developing research proposals, and undertaking studies which are submitted to them (for example, The National Institute for Health Research (NIHR) www.nihr.ac.uk).

People with experience of mental health issues are increasingly becoming involved in research, with benefits for research processes and for themselves [27, 28]. However, involvement in the early stages of research is not widespread. Only 10% of papers identified in a bibliometric review of public involvement in health research involved the public in identifying or prioritising research [29], and fewer than 20% of researchers consult people with experience of mental health issues at the early stages of conducting mental health research. In addition, their views are rarely considered in identifying research, and only occasionally included in defining the research question [18]. If they become involved before the research starts, people with experience of mental health issues can change the focus of a study to increase its relevance, thus improving quantity and quality of data collected from mental health patients [30, 31]. Consistently, people with experience of using mental health services report that they feel unable to influence research once it has begun [2, 5, 32].

It has been suggested that limited public involvement in research prioritisation may be linked to uncertainty about how to effectively involve people, including people with experience of mental health issues, in prioritising research and a perception that the process is cumbersome and complicated [10]. Researchers report a lack of training, skills, time and understanding as barriers to their role in involving people who use mental health services in their research [18, 33]. Approaches to effectively engaging people in setting the research agenda should acknowledge the rationale for involvement and ensure they understand the distinctions between research and service delivery [34]. A collaborative approach to research prioritisation, which encourages joint ownership by those affected by the issues being discussed, has been successful [2, 35], as well as feeling that the topics under discussion are relevant. The engagement of people with experience of mental health issues is stronger when those involved believe they are able to shape future research and practice [2, 33, 36].

As a group, the members reflected upon the value and benefits of the engagement of people with the experience of mental health services in order to identify research priorities for the group.

### Aim of this study

The aim of the workshop was to provide up-to-date evidence to inform the types of research the SUCPRDG could take forward over the next few years.

### Objectives

To meet the above aim we agreed to:

1. Review the literature around the involvement of people with lived experience and their carers in mental health research
2. Hold a workshop ‘Getting Involved in Research: Priority Setting’ with people with a lived experience of mental ill health, and their carers, to identify research priorities
3. Prepare a research paper outlining the results and the process for the benefit of others, and make this available as an open access publication
4. Demonstrate an inclusive collaborative approach in developing and identifying research priorities for people with experience of mental health services.

## Methods

In order to identify the research priorities for people with a lived experience of mental health services, and their carers, we chose a qualitative nominal group technique as this was deemed to be the most appropriate way to gather a collaborative view. It has been suggested that this technique is more productive and collaborative than other group techniques, and can contribute towards some creative data being gathered [37], whilst, providing the group with a structured method of brainstorming that encourages contributions from every member of that group.

The normal group technique is particularly suited to this research study as previous research has highlighted the power imbalance that can occur when professionals debate with members of the general public. This method promoted an equal voice, allowing every member to fully participating in group discussion, even those members who might not give their views in other group structures. It is also a useful method when some or all group members are new to the group, although a few members had already experienced this form of research [38], this was the first time this particular group had come together.

Following on from a short introductory session, participants were divided into three groups, who visited 6 workstations in rotation. Base around the main theme of that workstation, at each ‘workstation’, participants brainstormed and generated research ideas (as a form of mind mapping/collaborative activity). With every member of the group being given the chance to voice their ideas out loud, which was then recorded by the scribe. Each workstation took a similar format where the facilitator introduced the workstation theme and, along with the scribe, encouraged participants to discuss and generate ideas. These ideas were distilled as the discussions progressed and refined into research questions or ideas to be taken forward. Taking direction from the participants, the ‘workstation’ scribe transferred the research questions and ideas onto a flipchart. Upon completion of the workstation phase, three voting rounds were then held in order that the final research priorities could be declared and ultimately taken forward as suitable research topics by the RDG.

### Participants

Invitations to attend the “Getting Involved in Research: Priority Setting” workshop were sent out to people with a lived experience of mental ill health and their carers (via the SUCPRDG mailing list). The RDG has a Wales wide membership of people with a lived experience of mental ill health and their carers with an interest in mental health research. Personal invitations were also sent to 4 members selected through open opportunity advertisement from the Involving People Network,^1^ where members access the latest opportunities for involvement in research across Wales. A wide range of training and support is offered (with paid expenses and payment for time), together with regular newsletters and bulletins, support and guidance. Open invitations, along with details of the event, were also posted on the Mental Health Research Network website.

Participants were provided with details of the research and the workshop via email, and verbal consent was taken at the start of the workshop, of which 29 participants registered interest across Wales. An information pack was prepared by Hafal, using user friendly language, which was given to participants at the start of the workshop. The information pack was designed to help the workshop participants gain greater understanding of the reason for hosting the event, the structure of the day, the mental health themes under discussion (these had been decided by members of the RDG from amongst their membership). This information pack also contained background information about the structure and purpose of the RDG. The workshop took place from 10.00 until 15.30 on Tuesday, 14th May 2013, at Kegie Building, Caerleon Campus, University of South Wales, Caerleon, Newport. Out of the 29 participants that showed initial interest in the project, 25 (11 males and 14 females) participants went on to attend the workshop, in which 5 were mental health professionals, 20 were people with a lived experience of mental ill health, of which 3 were also carers. As it is not the purpose of this research study to analyses participant’s demographic data, to stay within the set ethical boundaries, apart from noting that all participants lived in Wales and participants gender, no other demographic data were collected.

Health and Care Research Wales (formerly NISCHR CRC) supported people with a lived experience of mental illness and their carers to attend by covering their travel and subsistence expenses and by offering payment for time for their involvement on the day, in line with Involving People Network guidelines.

### Workshop proceedings and data collection

The workshops were designed to help and encourage participants to be able to give their views and opinions about suitable priorities in relation to mental health research. Participants were introduced to the workshop aims, and the organisational format, by members of the RDG. This was an informal day where participants could make their views known and speak openly about research issues that were important to them. The role of moderator (s) or group facilitator (s) is critical, ‘especially in terms of providing clear explanations of the purpose of the group, helping people feel at ease,’ and facilitating genuine and purposeful interaction between group members [39].

Prior to the event, participants received an information pack, containing details about the workshop and the aims and nature of the event. It was important to participant particitation, that this pack to highlighted the open nature of the workshop, including a reminder that everyone within this study is of equal standing. This it was hoped would would be the first step towards increasing involvement and belief in the process. To reenforce the principle of everyone is equal, participants helped to set up the room, and other practical elements of the workshop, working together in partnership to achieve the group aims and to feel part of the event.

The workshop started with a plenary session where participants were again provided with the workshop aims and the organisational format. Following on from this, participants were split into three similar sized groups, and each group was given the opportunity to spend 30 min at each of the 6 individual workstations in turn. The workstations asked for different views on some of the main issues surrounding mental health research, with those issues being identified by the Research Development Group. The ‘workstation’ themes were:

- Workstation 1 – Treatment and Recovery
- Workstation 2 – Education and Higher Education
- Workstation 3 – Stigma and Attitudes
- Workstation 4 – Support Services / including Crisis Support
- Workstation 5 – Employment
- Workstation 6 – Other (Any topic not covered by the other 5 workstations)

Each of the workstations were organised around a workstation ‘facilitator’ and a workstation ‘scribe’. The ‘facilitators’ and ‘scribes’ were experienced members of the RDG apart from a small team (three people) from the Time to Change Wales campaign, who facilitated the ‘Stigma and Attitudes’ workstation.

When participants had visited all of the 6 workstations they were given an opportunity to vote for the research ideas generated. The research ideas were written-up by the individual workstation scribes onto large flipchart paper, and then placed around the room so that participant voting could take place.

## Results

A substantial number of research ideas were generated; 120 separate research ideas generated across the 6 different workstations. With 33 different research ideas, Workstation 1: ‘Treatment and Recovery’ had the highest number of different ideas generated by participants.

The first round of voting was achieved manually, where participants were given 2 spots per workstation, (12 in total) to place on the flipcharts identifying their favoured ideas. The second and third round of voting was achieved using handheld voting technology known as Turning Point.

The research questions and ideas that the participants generated at the workstations are listed in Tables 1, 2, 3, 4, 5 and 6 below.

**Table 1:** Workstation 1 – Treatment and recovery

How has the (Mental Health) Measure affected treatment in primary care?	
How do we measure the effects of the (Mental Health) Measure?	
What treatments are available to aid recovery? How can we help service users and carers to know about a full list of treatments and their effectiveness for different conditions?	
How do available treatments differ in different areas? Is there a league table and how can service users and carers access this?	
How does the Exercise Referral Programme affect/differ people with different Mental Health diagnoses?	
How does exercise make people with mental health problems feel better? And, How does it work for different diagnoses?	
Are some sports more effective (as treatment for depression) than others? And if there is why aren’t service users aware of this?	
How is information regarding informed choice disseminated to people with mental health problems?	
Is the choice of treatments available for service users actually an informed choice?	
How do service users select their treatment? Is there really any choice?	
How is the long term effectiveness of treatment measured?	
How do treatments change over time?	
Research that looks at the Geographical comparison of services provided through primary care under the Mental Health Measure.	
Is there any research that looks for a correlation between the ability to access services and their effect on recovery?	
How are carers supported during the recovery of the person for whom they care? Are they supported to embrace their changing role at this time?	
Are services fragmented with several different specialist services or are there different specialists in one team? Are crisis teams really integrated within Community Mental Health Teams?	
What are the effects on service users of having to deal with lots of different people, maybe in several different teams?	
What is the cost effectiveness of many different services being involved with one service user?	
Would it be possible to achieve coordination of services?	
What support is available in long term recovery?	
Are systems geared towards crisis rather than prevention?	
Are there specialist services for mental health problems seen as ‘less serious?	
Does diagnosis include an examination of physical problems which might cause mental ill health?	
Can ‘therapy’ be a cause of mental health problems?	
How can people with mental health problems develop their own support networks?	
Can Health and Safety regulations affect recovery? If so how?	
How do service users use self-management to realise their expectations and become empowered?	
How do the following affect service user experience: Information	
Whole person being treated	
Alternative treatments	
Working in partnership	
The effects of diagnosis on recovery	
How do service users inform professionals that recovery is possible?	
How do professionals ‘recover’ with service users?	
Once diagnosed, do service users feel that they are ‘labelled and forgotten’?	
Do service users feel that they receive adequate services and timely treatment?

**Table 2:** Workstation 2 - Education and higher education

How can we research into ways in which knowledge can assist in recovery, and how knowledge becomes power in mental health?	
There is a cost associated with engagement in learning. How can learning be made accessible to all service users?	
What efforts are being made to promote understanding of mental illness through education from an early age in infant, primary and secondary schools?	
What are the elements of education as a vehicle for treatment and recovery?	
How can education provide a ‘centre of gravity’ for recovery?	
Comparison of educational methods to promote recovery (e.g. full time, part time, formal, community based, informal).	
How is it possible to find out what support services are available for people with mental health problems and how do they find out how to access these, particularly if their condition is undiagnosed?	
Comparison of the benefits for recovery of accessing or re-accessing learning against not accessing learning.	
What support goes hand in hand with educational opportunities for service users as a model for best practice (e.g. mentoring)?	
How can community based learning be integrated with Campus based learning? What support is available to help this process?	
How is it possible to choose the most suitable of all the different routes into education?	
Is it possible to quantify the reduction in the level of ‘sick days’ directly attributable to education?	
Is there any provision within educational establishments for course work and assessments to reflect the possible effects on performance due to cyclic events in the mental health of service user students?	
How does ‘disclosure’ of a mental health problem affect access to learner support in education?	
What provision is there within different communities for service users to access both academic and non-academic learning?	
How can service users progress from project/community provision to Higher Education? Is progression both horizontal and vertical for the learner?	
In what ways do service users value education e.g. as a social skill, by achieving social interaction, through progression, through certification?

**Table 3:** Workstation 3 – Stigma and attitudes

How much money is spent on research into stigma and is this cost effective?	
What training do GPs receive to recognise and combat the effects of stigma?	
What are the attitudes of GPs, nurses and other NHS staff towards Mental Health service users? And do they receive training to combat the effects of stigma (their own and that of others)?	
How can attitudes of all staff within GP practices towards mental health service users be improved?	
Do service users experience ‘self-stigmatisation’?	
Is there enough initial support and information? What would be most helpful after initial diagnosis?	
Are labels useful? Is it possible to change the negative effect of labelling? Is it possible for service users to create their own (positive) labels?	
How can service users be provided with hope and direction to prevent self-stigma?	
Build a data bank of positive stories, in particular where negative labels were turned into positives.	
How can families of service users be helped to combat their lack of understanding, and be educated to recognise and react positively to the triggers and symptoms of mental distress?	
What causes people to have preconceived ideas about mental health? How can these be changed?	
Within family units, what causes people’s reactions to their family member who is experiencing mental illness? Is this different from their attitude to a stranger with mental illness?	
How much do the media affect people’s attitudes to mental illness?	
What terminology and labels are commonly used to describe people experiencing mental illness?	
What understanding do people (adults and children) have of mental illness and the effects of the labels used to describe it?

**Table 4:** Workstation 4 – Support services/crisis support

What do professionals consider makes a crisis?	
Who makes decisions around services and service consistency?	
How are service user mentors and peer supporters involved with support services?	
How can service users teach professionals?	
Are there good models of partnership working between service users and groups of professionals?	
How are pre-crisis situations recognised?	
Is it possible for education to be prescribed as treatment?	
What are the attitudes of home-treatment workers?	
Do service users decide where and why they are treated in certain ways. Do they also have a say in the length of their treatment?	
Who decides what is a crisis? How does funding affect that decision?	
How do service users know what services are available? Is centralised information available i.e. a one stop shop?	
Does location affect what support is available?	
How is the quality of support measured?	
Why are there ‘reactive’ crisis teams but not ‘preventative’ crisis teams?	
What strategies are available to prevent crisis situations arising?	
What options are available during a crisis and what works?	
What support is out there and what are the criteria necessary to obtain support?	
Why are ‘non mental health’ professionals not educated in mental health issues?	
Do support services make you ill?	
What support do carers receive?

**Table 5:** Workstation 5 – Employment

Can work be therapeutic? What is the role of employment in promoting physical and mental well-being?	
What are the positive contributions of employment to mental health e.g. focus, dedication?	
Is meaningful and appropriate work available to service users who may require an adapted environment, flexi time, specialist support etc.?	
What are the challenges of returning to work?	
What effect might the Health and Safety Act have on service users wishing to return to work?	
Is it possible to make a business case for employers to take on people with mental health issues?	
How can an ‘institutionalised’ service user be persuaded to return to work?	
How can employers be educated and encouraged to employ people with a mental health problem?	
Why do some employers have preconceived ideas that people with a disability will be less productive than others?	
Can people with mental health problems be of benefit to an employer?	
What support is there for people with mental health problems to become self-employed?	
Can service users going into employment be persuaded that, even if they lose benefits, they will increase their self-esteem?	
Is ‘transitional support’ available for people with mental health problems accessing or returning to employment? Is training and support provided for supporters?	
What are the positive effects of having a person with mental health problems in the work force?	
Is it possible to provide ‘Ambassadors’ to link employers with service users and to help overcome barriers to employment?	
Is there a difference in the ‘barriers’ to employment experienced by people with different types of mental illness e.g. bipolar disorder, schizophrenia?	
Is there discrimination against people with ‘sectionable’ illness in voluntary roles as well as in paid employment?	
What can be done to help service users achieve employment in their chosen field e.g. IT training etc.

**Table 6:** Workstation 6 – Other (any topic not covered by the other 5 workstations)

How can service users become involved in research? Can they obtain training and help with the specialist language of research? Can research be made accessible to non-specialists?	
How can carers of service users become involved?	
Are activities available for carers out of the normal 9–5 h of the working day?	
How do service users get support to gain motivation and purpose? How frequently do ‘purpose’ and ‘motivation’ feature in recovery plans?	
What proportion of funding for mental health care goes to the voluntary sector? Research into the activities of the police and prison workers in the care and treatment of people with mental health problems. The prison population could be useful for research in this.	
Are Welfare Reforms having a negative impact on mental health and thus making people more ill?	
The effects on mental health of childhood abuse	
How does the intervention of the law, including police and probation, impact on people with mental health problems?	
How do people help themselves? How do they benefit? What characteristics are needed for successful self-help?	
How is funding allocated between services?	
How can service users obtain support to maintain momentum towards recovery?	
Should the place of the pharmacist as expert over the GP be acknowledged and should the pharmacist be used more widely to explain drug treatment, side effects etc.?	
How widespread in treatment is recognition of the whole person, physical, mental and spiritual?	
Case studies – What do people do to aid their own recovery? What are individual trigger points? How do people learn from experience? How common are ‘circles of support’?	
How can research show that everyone is of value	

### First round of voting

The first stage of voting, called the “spotlight” voting round, saw each participant given 12 coloured sticky spots - 2 votes (sticky spots) per workstation. Voting was undertaken manually and participants were asked to vote for their top two research ideas from each of the workstations, by placing a coloured “spot” alongside the individual workstation ‘ideas’ sheet (s). The following research priorities were identified within each workstation below:

#### Treatment & recovery

How do people with a lived experience of mental ill health develop their own support networks?

How are carers supported during the recovery of the person that they care for?

#### Education & higher education

What support goes hand in hand with education opportunities?

How can education provide a centre of gravity to recovery?

#### Stigma & attitudes

How much does the media affect people’s attitudes to mental illness?

Undertake research in schools and colleges to find out what training and support they provide?

#### Support services and crisis support

Why are they crisis intervention teams but not crisis prevention teams?

How are people with a lived experience of mental ill health mentors involved in support services?

#### Employment

Can people with mental health problems be of benefit to employers?

How are skills provided to a person with experience of mental ill health so that they can achieve their aims?

#### Other

How do people with a lived experience of mental ill health gain support to maintain momentum towards recovery?

Are welfare reforms making people more ill?

### Second and third stages of voting

Participants were given individual voting handsets and had the opportunity to vote anonymously for the priority that they would most like to see researched by the people with a lived experience of mental ill health and carer RDG. In a very close vote, the research priority to receive the most votes was:-

*Undertake research in schools and colleges to find out what training they provide to their students regarding mental health and can this training be improved?*

The third vote was then undertaken to find the priority which participants felt could most easily be translated into an immediately ‘doable’ research subject/application for research funding by the RDG. After another close vote the research priority to receive the most votes was:-

*How can education provide a ‘centre of gravity’ to recovery, including support? e.g. mentoring?*

In addition, participants engaged in a notable discussion over the research priority:

*‘How are carers supported during the recovery of the person for whom they care?*’

It was widely agreed by the participants that, as there were a smaller number of carers present at the workshop, and this influenced the voting in favour of people with a lived experience of mental ill health. It was therefore agreed that this research priority should also be taken forward by the RDG as a matter of priority for the immediate future.

## Discussion

The nominal group technique [37] was a useful approach to take when the goal of the activity was to generate ‘user’ led research, as it is a collaborative approach to data collection. All participants were given the opportunity to discuss their views, although there may have been some people who disliked talking in a group. The role of the facilitator, within the confines of the nominal group technique, is to encourage group discussion, allow participants the space and time to think, to give every member of the group a voice, and to clarify issues raised, so it is important that they have experience in managing groups. The 6 workstations asked for different views on some of the main issues in mental health research, identified by the research development group. The themes of each workstation will now be discussed in the sections below.

### Treatment and recovery

This workstation-generated discussion around the current research on treatment and recovery in mental health care and what priorities people with a lived experience of mental ill health, and their carers, could identify for future research. See Table 7 above for the full list. It is evident that much of this discussion focused upon key areas for research including mental health recovery, law and access to services including crisis prevention and other treatments. This indicates that there is a lack of information still for people with a lived experience of mental ill health and their carers around how they might get the best out of mental health services [2, 5, 32].

**Table 7:** Benefits of engagement with people with experience of mental health services

Benefits to the research	Benefits to the person with a lived experience of mental ill health
Anyone can experience mental ill health	Reduces the effects of stigma
First-hand experience	Acknowledges individual experience
Other life skills other than mental health experience	Self-development and education
Acknowledges the lived experience whilst gaining access to the knowledge associated with that experience	Feel valued and able to contribute
Services are more relevant and not wasted	Empowering and builds self esteem
Make informed decisions for the future that will benefit all based on this type of direct input into research projects	Therapeutic for all participants
People with lived experience have as much (if not more) insight into the problem / condition which may be overlooked by the academic	Knowledge development for all participants
To better ensure that services meet needs	Addresses legal and political rights

### Education and higher education

Participants at this workstation generated a great deal of discussion around the issue of access to education and how education might improve mental health recovery. A number of issues came from this, including how might such issues be addressed to improve access? Participants at this workstation spent a great deal of time discussing the importance of education for mental health recovery, and how this information might be made more available to the public and local community.

### Stigma and attitudes

At this workstation participants identified the importance of providing training and education to the general public, particularly young people (and more specifically the media), to combat the issues of stigma, labels and attitudes towards people with mental ill health. A number of discussions at this workshop were strongly linked to the education workshop, in that it identified the need to provide adequate resources to inform and encourage public support and understanding of mental ill health in order to effectively combat stigma and discrimination.

### Support services, including crisis support

Participants at this workstation identified the importance of ‘partnership working’ in order to be able to effectively identify a ‘crisis’. And, how a ‘partnership approach between a person with a lived experience of mental ill health, their carer (s) and health professionals could all work as a team in providing support for people who are in crisis. The recognition of divergent types of support were identified including peer mentors and educating the public promoting the idea, that people in the local community can also provide support during a crisis. The question *“do support services make you ill?”* offered the opportunity for people to identify what adequate support services might look like if they were to be truly therapeutic and recovery focused.

### Employment

In this workstation people identified many of the key issues to do with access to employment, including support, training and access to opportunities for people with experience of mental ill health and how they can be supported to return to work. Finding the balance between the therapeutic effects of work and the expectations of others (which could be responsible for someone returning to work too soon) were discussed in some detail, including the suggestion of work Ambassador to help with the transition back into work.

### ‘Other’ – any topic not covered by the other 5 workstations

This workstation covered areas of mental health service and practice that had not previously been discussed at any of the previous workstations. The idea behind this was that participants should be given the opportunity to discuss any other issue that was important to them. There were a large number of research questions generated at this workstation and this certainly suggests that people with experience of mental health services are well able to generate and prioritise areas of research that health professionals may not be considering. This includes access to research e.g. research opportunities and research findings, becoming more involved in research, the types of support and resources available for becoming more involved in the development of mental health services and the education and training needs of children and young people in school. This suggests that the participants felt that it was important to break down stigma and discrimination and to support young people to have better mental health skills. Participants at this workstation stated that information on how to access support and services for mental ill health may still be lacking; especially in lack of access to NICE recommended care and treatment and this area in particular urgently requires further research.

### Main outcomes

The generation of so much information required a voting system to be put in place, so that priority goals for ‘user’ research could be set. The nominal group technique helped us to gather enough information to do this, and collaborate on what were the most important issues for people with a lived experience of mental health research [30, 31].

Two main research priorities were identified and these were developing the knowledge of mental health issues amongst school-aged children and developing education as a tool for recovery for example by peer support. In addition, participants engaged in a notable discussion over the research priority: ‘How are carers supported during the recovery of the person for whom they care?’

The main findings from the priority setting research day were taken up by the RDG to support the group in its research activities, including the generation and development of research funding applications for ‘user’-led research.

In addition to this, the research findings were used to support a funding application by the Time to Change Wales campaign, in support of their ongoing anti-stigma programme. They successfully secured nearly £500,000 from the Big Lottery, Cymru, Fund to increase young people’s awareness and understanding of: mental health problems, stigma, discrimination and social isolation experienced by those with mental health problems. This new Young Persons Programme (due to commence at the end of 2016) builds on the existing Time to Change Wales campaign, which was launched in 2012, to reduce the stigma and discrimination faced by adults with mental health problems in Wales.

#### Limitations

This paper discusses a qualitative inclusive approach to research that attempted to ensure that all voices are heard. This can be difficult in a research environment and it is inevitable that some voices might be lost. The facilitation of the groups is an important feature of the nominal group technique, which requires patience and experience. All facilitators were experienced in group management so that every effort was made to capture all ideas; the generation of so many ideas supports this view. The voting stages also provided the opportunity for people to anonymously choose the priorities for research, without the interference of professional advisors or managers, who might have their own agendas for research [2]. We feel that the limitations of this research were kept to a minimum but acknowledge that some people may not have been able to participate as much as they would have liked.

## Conclusion

This paper sets out to offer an important contribution to mental health ‘user’ research. The aim was to discover the research priorities of people with a lived experience of mental ill health and their carers. By providing an inclusive and supporting approach it is more likely this was successfully achieved at the workshop. Two main research priority areas were identified:

1. ‘How to overcome stigma and discrimination by educating young people to better manage their own mental health and wellbeing?’
2. ‘How best to develop education as a tool, and support for recovery for people with a lived experience of mental ill health?’ including for example how best to take forward peer support and work Ambassadors’. In addition, participants engaged in a notable discussion over the research priority: ‘How can carers take up the reigns of their own life as the person they care for is in recovery?’

The RDG is confident that it has addressed the research outcomes by identifying, creating and co-producing ‘user’-led research in collaboration with people who have a lived experience of mental ill health. This paper is the result of continued collaboration between members of the Service User and Carer Research Partnership, based in Wales and supported by the NCMH and Health and Care Research Wales.

Since this research study ‘Time to Change Wales’ have used these findings to apply for and be granted a funding grant from the National Lottery. Although the details of the grant application is not known, in accordance with this study’s strong ethical stance, the report has to highlight a possible conflict of interest.

## Abbreviations

GP: General practitioner
MHRN-C: Mental health research network cymru
NCMH: National Centre for Mental Health
NHS: National health service
NIHR: National Institute for Health Research
NISCHR: National Institute of Social Care and Health Research
NISCHR CRC: National Institute of Social Care and Health Research Clinical Research Centre
RDG: Research development groups
RRG: Registered research group
SUCPRDG: Service user and carer partnership research development group

## Footnotes

1 Funded by Health and Care Research Wales, the Involving People Network brings together members of the public from across Wales who are interested in working with researchers to improve treatments and care. Involving People Network members have access to the latest opportunities to get involved in research in Wales.
